# Staff costs of hospital-based outpatient care of patients with cystic fibrosis

**DOI:** 10.1186/2191-1991-1-10

**Published:** 2011-08-03

**Authors:** Helge Hollmeyer, Jonas Schreyögg, Ulrich Wahn, Doris Staab

**Affiliations:** 1 Department of Pediatric Pneumonology and Immunology, Charité University Medicine Berlin, Germany; 2 Institute of Health Economics and Health Care Management, Helmholtz Zentrum Muenchen, Germany

**Keywords:** Cystic fibrosis, outpatient service, cost analysis, micro-costing, activity-based costing, respiratory tract colonization, lung function testing, analysis of variance, multivariate analysis

## Abstract

**Background:**

This study identified per patient resource use and staff costs at a cystic fibrosis (CF) outpatient unit from the health care provider's perspective.

**Methods:**

Personnel cost data were prospectively collected for all CF outpatients (n = 126) under routine conditions at the Charité Medical School Berlin in Germany over a six month study period. Patients were grouped according to age, sex and two severity categories. Ordinary least squares regression analysis was performed to determine the impact of various independent variables on personnel costs.

**Results:**

The mean staff costs were €142.3 per patient over six months of outpatient service. Services provided by physicians were the biggest contributor to staff costs. Patient age correlated significantly and negatively with mean total costs per patient.

**Conclusions:**

Age of patient is a significant determinant of staff costs for CF outpatient care. For a cost-covering remuneration of outpatient treatment it seems plausible to create separate reimbursement rates for two or three age groups and to consider additional costs due to tasks carried out by physicians without direct patient contact. The relatively low staff costs identified by our study reflect a staffing level not sufficient for specialist CF outpatient care.

## Background

Cystic fibrosis (CF) is the commonest life-threatening genetic disease occurring in approximately 1/3500 white newborns [1]. This autosomal recessive disorder is characterised by chronic endobronchial bacterial infection and neutrophil mediated inflammation leading to progressive pulmonary deterioration, respiratory failure and premature death [2]. Other clinical consequences include pancreatic insufficiency, liver disease that may progress to cirrhosis, gut motility problems and elevated sweat electrolytes. CF-patients need an intensive, holistic and life-long treatment provided by specialist care [3,4].

The management of CF is an expensive commitment on the part of health care providers and those who fund them. Costs of care for patients with CF are rising as a result of new medical interventions that improve health status and life expectancy. In the United States of America (USA), the median age of survival for CF patients, i.e. the age at which half of the current population with CF would be expected to survive, has risen to 35.9 years in 2009, up from 27 years in 1985, 14 years in 1969, and 5 years in 1955 [5]. With enhanced screening and implementation of new therapies the predicted median survival for newborns in 2000 with CF is likely more than 50 years [6]. As a consequence of improved prognosis this complex inherited disease will have considerable implications on the resources required for case management.

In Germany, outpatient treatment for most of the 8000 CF patients is provided by about 110 hospital-based CF reference centres [7]. Given the complexity of the disease, the centres comprise variable numbers of specialist CF physicians and nurses, dietitians, physiotherapists and psychologists. These health professionals follow defined standards for the evaluation, monitoring and treatment of CF outpatients including surveillance of pulmonary function and bacterial colonisation status, early management of infection, education on inhalation and airways clearance techniques, assessment of and advice on nutritional status, as well as psychological support for patients and their families. Comparative data suggest that the care provided in CF reference centres substantially improves patients' well being and quality of life [8]. However, staff levels at CF centres in Germany are far below those recommended by the European Consensus Conference [9]. A recent study conducted by the German CF association found that only about 50% of the costs for hospital-based outpatient care are reimbursed by the public health insurance system, while the remaining costs are usually borne by the hospitals [10].

Several studies have been published on hospitalization costs or cost-of-illness of cystic fibrosis [8,11-22]. However, to date only few studies have conducted a detailed cost analysis for outpatients [8,9]. Moreover, most studies calculating the costs of CF are based on a gross-costing approach. Only two studies used a micro-costing approach in calculating costs of hospital and outpatient care, respectively [15,23]. Such information is important for understanding the true costs of all components of CF treatment and underlying reasons for variation in costs. The present study provides a detailed activity-based analysis of the actual use of health personnel, which is usually perceived as a major cost factor in outpatient care. Given the identification of wide interindividual variation in disease severity and hospitalization costs for patients with CF [15,17,18,20,22], we hypothesized that staff costs for outpatient care vary with patient-related characteristics.

To aid public health policy makers in their efforts to ensure a financial coverage for CF outpatient care that is commensurate with the actual expenses, we undertook a prospective study with the following objectives: (1) to identify per patient average resource use and costs for personnel at a CF outpatient centre from the health care provider's perspective; (2) to assess whether outpatients can be grouped into cost homogenous clusters of patients according to sex, age group, and disease severity levels; (3) to evaluate the value of demographic and clinical variables in predicting staff costs.

## Methods

### Study subjects

Our prospective study on health personnel utilization and determinants of staff costs in hospital-based outpatient CF care was carried out between January and June 2004. All patients (n = 126) who attended the CF outpatient unit of the Department for Pediatric Pneumology and Immunology at the Charité Medical School Berlin in Germany during this period were enrolled. We assumed that this time frame provided a representative mix of CF patients undergoing routine check-ups at the outpatient centre.

### Data collection

The following data were collected for all patients that visited the CF outpatient centre during the study period: patient age, sex, clinical parameters, and use of staff. The clinical data included respiratory tract colonization by *Pseudomonas aeruginosa*, respiratory insufficiency and forced expiratory volumes in one second (FEV1). Personnel costs directly incurred by each outpatient were prospectively collected for all visits under routine conditions by using a micro-costing approach. There are pros and cons for using micro-costing versus gross-costing approaches. An important advantage of micro-costing is that it considers certain cost components in greater detail. Gross-costing has the advantage of being less expensive than micro-costing, but it is less sensitive and often underestimates certain costs, e.g. personnel costs for resource intensive patients. Thus, we believe in this context it is important to provide a high sensitivity of cost estimates and therefore used a micro-costing approach [24,25]. For the personnel involved in the treatment at our outpatient centre we measured the exact time devoted to an individual patient on an activity-per-patient basis. To measure the time spent for each predefined activity, all staff members at the outpatient unit were equipped with stopwatches and required to document the exact time devoted to each patient. Prior to data collection, all staff members providing services at the outpatient centre were interviewed to determine principal activities administered by different health professions using activity-based costing (ABC) methodology. ABC can be regarded as an instrument of the micro-costing approach. The time-driven ABC methodology that we used allows organizations to determine actual costs according to the (health care) activities that originate these costs [26-30]. The actual resource consumption is estimated by taking into account how much time it takes to carry out each kind of activity and the costs per time unit associated with the services. ABC is also a practical tool to identify cost drivers, to evaluate possible resource or practice changes in the health care process, and to identify areas where the quality and efficiency of care can be improved [31-33].

Resource utilization not attributable to an individual outpatient visit and therefore not varying among patients, such as staff meeting or interdisciplinary coordination, was not measured. For physicians and nurses, all measured activities were only administered in direct contact with the patient. All professions (physicians, CF nurses, dietitians, physiotherapists and medical assistants who are either secretaries or doctors' assistants) were working only part-time in outpatient treatment and had to devote the largest amount of their working hours to inpatients with CF hospitalized at the same health care facility. Thus, we considered each profession with its actual working capacity in the outpatient unit. Services provided by psychologists and social-workers were not measured because of a lack of personnel. These professions provide services at the outpatient centre only in emergency cases. Informed consent was not required because there were no interventions affecting either physician's treatment decisions or the patients.

### Cost calculation

Staff costs for outpatient care were calculated in Euros (€) for the year 2004. Real staff costs were calculated from the health care service perspective by multiplying the collected time data by the wages of the respective care provider professionals. Specifically, the actual time measured for each activity in minutes was multiplied with the labour costs per minute (unit costs) of work for the respective health profession from the perspective of the health care provider. The unit costs that we used in our study are based on the gross labour costs ("Arbeitgeberbrutto") of all personnel employed within a specific wage group according to the German public service salary scheme ("BAT"), which encompasses social security and pension contributions as well as sick and annual leave. We used the average gross labour costs for each occupational category as identified by the study of Eidt-Koch in a representative university hospital [23].

### Classifications

Patients were classified according to age (> 11 years, 11-18 years, > 19 years), sex and disease severity. Based on the classification suggested by the CF Foundation, patients were grouped by three severity levels of their respiratory function; mild, moderate, and severe corresponding to FEV1 of ≥ 70%, ≥ 40% but < 70%, and < 40%, respectively. A three level severity index was also applied to all patients based on *P. aeruginosa *respiratory colonization and respiratory insufficiency. Patients with pulmonary hypertension and global respiratory insufficiency were classified as severe; patients with chronic *P. aeruginosa *colonization of the lungs but without pulmonary hypertension and respiratory insufficiency were classified as moderate; and patients without chronic colonization (*P. aeruginosa*) were classified as mild.

### Statistical analysis

Using the two severity categories and the age related classification, we investigated whether patients could be grouped into homogenous clusters with respect to mean costs, and assessed the variance between each group (F-test). Ordinary least squares (OLS) regression analysis was performed to determine the impact of FEV1, diagnosis-related severity, age and sex on personnel costs for outpatient management and frequency of visits. The natural logarithm of costs was used as the dependent variable for the regression model because the distribution of "total costs per patient" was skewed. Descriptive statistics and analysis of the residuals were conducted to determine the adequacy of the regression analysis. For the variance and regression analysis, the lung function was included as the highest individual FEV1 value of all spirometry results during the study period. All statistical analyses were performed using STATA (version 10).

## Results

### Study population

The study included 126 patients visiting the outpatient centre during the study period. Their demographic and clinical characteristics are shown in table 1. Of these subjects the median age was 18 years while ages ranged from nine months to 55 years. The lung function was abnormal (FEV1 < 80%) in the majority of adults (80%) and in half of the younger patients (< 18 years). Twenty three patients were under six years of age and, thus, spirometry data were not available for them. Consequently, 103 patients were considered for the evaluation of FEV1 as a predictive factor for the variation of staff costs among patients. The mean FEV1 was 63% among this group, and the impairment of the respiratory function was mild in 40 patients (FEV1 ≥ 70%), moderate in 41 patients (FEV1 < 70% but ≥ 40%), and severe in 22 patients (FEV1 < 40%). With respect to the diagnosis-related severity index, 12% of the patients had severe disease, 52% moderate and 36% mild diseases.

**Table 1:** Study group characteristics, outpatient visits and mean total staff costs per patient per six months, and results of variance analysis for demographic and clinical parameters.

Variable	n (%)	Outpatient visits	Total patients' staff costs in € (± SD)
**Age**			
children (aged < 11 y)	36 (28.6%)	2.9	181.9 (± 124.3)**
children aged ≤ 1 y (n = 9)	9 (7.1%)	4.4	296.9 (± 169.3)**
children aged > 1-6 y (n = 18)	18 (14.3%)	2.2	147.1 (± 74)
children aged 7-10 y (n = 9)	9 (7.1%)	3.0	136.7 (± 86.2)
adolescents (aged 11-18 y)	30 (23.8%)	3.2	143.7 (± 89.1)
adults (aged 19-55 y)	60 (47.6%)	2.9	117.9 (± 82.1)**
			
**Sex**			
males	63 (50%)	2.8	135.8 (± 96.4)
females	63 (50%)	3.1	148.9 (± 104.7)
			
**Lung-function-related severity level**			
mild (FEV1 ≥ 70%)	40 (38.8%)	2.2	113.5 (± 71.2)
moderate (FEV1 < 70% to ≥ 40%)	41 (39.8%)	3.5	132.6 (± 80)
severe (FEV1 < 40%)	22 (21.4%)	3.1	143.8 (± 110.7)
			
**Diagnosis-related severity level**			
mild (absence of *P. aeruginosa*)	45 (35.7%)	2.7	162.4 (± 121.5)
moderate (*P. aeruginosa *colonization)	66 (52.4%)	3.2	128.4 (± 82.1)
severe (pulmonary hypertension and global respiratory insufficiency)	15 (11.9%)	2.9	143.2 (± 101.1)

**p ≤ 0.01 (F-test for each group vs all remaining patients).

During the six months study period, patients attended the outpatient centre three times each on average, ranging from one to twelve visits. Of all outpatients 29% had only one visit, while 30% attended the CF centre twice and 41% three or more times. A group of 20 patients had six or more outpatient visits during the study period, of which 14 patients were "moderate" according to the diagnosis-related severity category.

### Cost results and resource use

The mean total staff costs per patient amounted to €142.3 over six months of outpatient service, ranging from €22.1 to €669. Validation and plausibility checks revealed that cost data were about 80% complete. The costs according to patient age, sex and clinical characteristics are reported in table 1. Among the patients with total costs above €270 (n = 13), three patients (21%) had severe levels according to both severity categories, five (38%) were infants (≤ 1 year) while three (23%) were older than 18 years. For infant patients (n = 9) the average cost was €296.9. Table 2 shows in detail mean total costs and resource utilization per patient for the different health professions and specific activities. Out of the total mean time spent per patient over six months, 37% was attributable to physicians and 30% to nursing staff. Resource use with respect to medical assistants, dietitians and physiotherapists was much lower accounting for 15%, 11% and 8%, respectively. During each outpatient visit, patients were treated by physicians and nurses, i.e. a total of 374 times, while the utilization of medical assistants, dietitians and physiotherapists was considerably less frequent (80%, 28% and 26%, respectively).

**Table 2:** Mean outpatient staff costs and resources used for a six months study period categorized by different health professions.

Health profession	Average gross labour costs per year (€)	Resources used (minutes)	Unit costs (€)	Mean Costs (€)	Median	SD
**Physicians (total)**	**74.135 **(BAT Ib)	**91**	**0.77**	**70.4**	**52.1**	**62**
Clinical history and physical examination		47	0.77	36.2	25.9	31.6
Consultation on diagnosis and therapy		44	0.77	34.2	26.7	37.6
						
**CF nurses (total)**	**42.730 **(KR. III)	**75**	**0.44**	**32.9**	**24.3**	**28.3**
Identification of medical conditions		18	0.44	8.1	7.2	8.0
Assistance to physician		19	0.44	8.4	13.5	13.2
Lung function test		12	0.44	5.1	4.4	4.9
Other nursing services		25	0.44	11.2	8.1	14.1
						
**Medical assistants (total)**	**40.833 **(BAT Vc)	**37**	**0.42**	**15.5**	**9.2**	**20.7**
Preparation of tests		6	0.42	2.4	3.2	9.3
Prescriptions and certificates		12	0.42	4.9	3.2	8.6
Accounting		20	0.42	8.3	3.0	17.0
						
**Dietitians (total)**	**50.007 **(BAT IVb)	**26**	**0.52**	**13.7**	**18.2**	**15.4**
Nutrition consultation		18	0.52	9.4	13.0	11.6
Information, documentation, organization		8	0.52	4.3	5.2	5.2
						
**Physiotherapists (total)**	**46.222 **(BAT Vb)	**20**	**0.48**	**9.8**	**9.3**	**16.0**
Physiotherapy		18	0.48	8.6	7.6	15.2
Information, documentation, organization		3	0.48	1.2	2.1	1.5

**Total**		**250**		**142.3**	**122.3**	**100.5**

### Analysis of variance

The breakdown of total costs per patient into the categorical severity models, sex and age groups shows the highest utilization of outpatient care to be among patients below 11 years of age, females, and patients with severe FEV1 and mild diagnosis (table 1). The difference between the average costs of the group of patients under 11 years and the average of the group above that age was statistically significant. Within the group of children (mean age = 4.2 years), the cost difference between the mean of infants (≤ 12 months) and the mean of children aged older than one year to 10 years was also significant (p < 0.01). The total staff costs per patient were higher for female (€148.9) than for male patients (€135.8), although statistical significance was not reached. The differences in total costs for the severity categories were also not significant.

### Regression analysis

As independent variables, the diagnosis-related severity category had logical correlations with age and FEV1, while FEV1 was correlated with age. Table 3 shows regression coefficients for each of the potential predictors of "total staff costs per patient" as the dependent variable. In the OLS-regression analysis, age showed a negative significant correlation, whereas sex, FEV1 and diagnosis-related severity indices were not statistically significant predictors. Only for adolescents did both severity categories demonstrate a significant association with total costs per patient (P < 0.05). Frequency of outpatient visits was correlated with low costs per visit, and younger patients tended to visit the outpatient unit more frequently.

**Table 3:** Results of ordinal (sex and diagnosis-related severity level) and linear (age and FEV1) regression analysis with 'Ln (total staff costs per patient)' as the dependent variable.

Independent variable	Standardized Coefficient (β)	Standard error	t-value	p-value
Constant	5.079	0.386	13.16	< .0001
Age	-0.018	0.007	-2.53	0.013
Sex	0.216	0.134	1.61	0.11
FEV1	-0.005	0.004	-1.33	0.185
Diagnosis-related severity level	-0.034	0.144	-0.24	0.812

R-square: 0.08; Adjusted R-square: 0.05; F-value: 2.25

## Discussion

In our prospective study on individual utilization of health care personnel, the average staff costs for six months of outpatient care were €142.3 per CF patient from a health care provider's perspective. Services provided by physicians were the biggest contributor to staff costs (48%). With respect to the impact of outpatient's demographic and clinical characteristics on staff costs, age correlated significantly and negatively with mean total costs per patient. Neither the diagnosis nor the lung function-related severity model were independent risk factors for high staff costs and seem to be inappropriate criteria for the classification of patients into homogenous cost groups.

The present study identified higher staff costs and frequency of outpatient visits among younger patients, especially infants. Increased resource use for infants is due to the intensive diagnostic workup which usually takes place during the first year of life. The high frequency of attendance among adolescents reflects the first infective respiratory exacerbation that usually occurs during this period of life. Irrespective of disease factors, younger patients require more time for social interaction by staff to explain and apply procedures. In contrast, older patients are more experienced with respect to self-management and many above 25 years are likely to have mild disease, thus not requiring a case management above the routine number of outpatient visits [8]. Moreover, inpatient admissions of older patients with more severe disease resulted in less frequent outpatient visits during the study period. It is therefore not surprising that adult patients caused lower staff costs than younger age groups.

According to our literature review, eight studies [8,10,13,14,17,18,21,22] estimated resource utilization for hospital-based CF outpatient services as part of total annual costs. The cost analysis of six studies was based on individual resource use under routine care [8,10,13,14,21,22], while two calculated annual costs from the aggregate perspective [17,18]. Only two studies examined different cost categories for outpatient treatment allowing the estimation of costs for health personnel [22,23]. Comparison of our findings with those of other studies addressing CF outpatient costs is not straightforward as the methodology used, perspective adopted, and health service utilization and organization vary. However, some common threads could be identified.

Eidt-Koch assessed the total resource use for CF outpatient care including costs for personnel, laboratory examinations, medication, overhead and infrastructure [23]. In addition, this study compared the evaluated costs with the actual reimbursement according to the official German Remuneration Scheme for Outpatient Care (EBM) and showed that about half of the costs are left to the CF centres suggesting that "alternative reimbursement schemes should be thought of" [23]. Specifically, Eidt-Koch's study explored determinants of costs by dividing 326 patients in seven CF centres in Germany according to their age, FEV1 values, certain co-morbidities, and bacterial lung colonization. For all disease characteristics but FEV1, outpatient costs differed significantly between the patient groups.

The utilization of personnel, estimated in Eid-Kocht's study by patients themselves during one representative month in 2006, showed somewhat higher staff costs than in our study. Of the €488 mean total costs per patient per quarter for CF outpatient care (including costs for laboratory tests but not drugs), Eidt-Koch found staff costs of €81.7 for all diagnostic and therapeutic activities performed by physicians (€42.4), nurses (€27.4), physiotherapists (€6) and dietitians (€3.2). Resource use for services provided by psychologists and social workers, not considered by our study, were estimated as only €1.2, respectively. Costs identified by our study were slightly lower (€71.2 per quarter) for similar activities and when extrapolated to the same time period, mainly because of fewer resources measured for lung function tests (€2.6 vs. €24 per quarter). The unexpectedly low resource use identified in both studies for diagnostic and therapeutic activities, which are essential in CF outpatient care, indicates that services were provided under enormous time pressure because of considerable under-staffing and parallel work load with respect to hospitalized patients with CF. Another study on hospitalization costs of CF that we implemented simultaneously at the same health care facility confirmed that all personnel involved in both inpatient and outpatient care had to apply by far the greatest part of their working time to the management of inpatients with CF [15]. In Eidt-Koch's and our study, outpatient care was provided by only 2.36 and 1.75 personnel per 100 patients, respectively, which is far below the level recommended by the European Consensus Conference (9.6-13.6 personnel). Therefore, staff costs for outpatient care would probably be much higher if CF centres were equipped with sufficient personnel who could then apply treatment in a manner as suggested by the European consensus.

Although not quantitatively measured by our study, physicians reported that the vast majority of their work time spent at our CF centre is used for the organization of outpatient care instead of direct patient contact treatment. Importantly, in Eidt-Koch's study, staff costs that were incurred for tasks without involvement of individual patients amounted to €134 per patient per quarter, which is far more than for activities with direct patient contact. These activities, carried out by physicians, include the organization and documentation of outpatient and home-based care, quality assurance measures, interdisciplinary coordination and continuous CF-specific medical education. Given that in Germany only about 50% of the actual costs for hospital-based outpatient service utilization are reimbursed [10,23], these additional activities without patient contact have to be considered for a cost-covering remuneration.

In a study conducted in the UK in 1990, outpatients' attendances were registered and time proportions were allocated to 51 individual patients [22]. Total costs per patient amounted to £677 per year with health personnel utilization by far the largest expenditure, accounting for 55% (£370), while 34% of the costs were attributable to medications. Of total staff costs the proportion incurred by each subgroup of health professionals differed considerably from our findings (34% for physicians, 33% physiotherapists, 25% social workers, 8% nurses, 1% dietitians). However, the results of this study are difficult to compare with ours because of differences in the methodology for measurement of resource utilization and in the organization of health services. Furthermore, the study population included only adults with a different ratio of mild to severe cases. Other studies measured total costs of CF outpatient care without specifying the proportion of personnel costs. In France, the annual costs of hospital-based outpatient treatment were estimated as between €354 [13] and €502 [14] in 2000/2001, based on different data gathering methodologies. Horvais showed that outpatient costs including costs for medication and home health care accounted for 88% of total costs for CF patients in France [14], where hospital stays have decreased in favour of home treatment [34]. In the USA, the mean overall costs of CF outpatient visits were retrospectively estimated as $1,500 per patient per year from a societal perspective in 1996 [18]. A study from Canada conducted in the same year found, with $899 annual costs per patient and $228 per clinic visit, much lower costs [8]. Compared to the average outpatient visits per patient identified in our study (6 per year), this number ranged from 3.3 to about seven visits per year in other studies [13,17,18,21,22].

The strength of our study lies in the detailed and long-term measurement of actual resource consumption at the individual level to calculate staff costs of outpatient CF care. To our knowledge this is the first study that used an activity-based costing methodology under conventional treatment conditions to measure staff costs. The distribution of patient characteristics were about the same as for the average CF population documented by the German CF patient registry [35]. This finding indicates that our results can be extrapolated to other CF centres in Germany. As difficult decisions about health care delivery and its funding have to be made, we believe that our bottom-up analysis provides reliable information to analyze the cost-effectiveness of and payment rates for CF outpatient treatment. However, the basis of unit costs for health professions has changed considerably since the implementation of our study. The costs determined in our study have therefore to be seen in the context of the BAT salary scheme at that time. For future negotiations on a cost-covering remuneration of CF outpatient care, only the resource use in minutes should be considered.

Limitations of our study include the potential underestimation of resource utilization because personnel had to simultaneously provide care for inpatients and outpatients. Staff may also have forgot to measure up to 20% of activities due to time constraints and emergency visits, according to plausibility checks. Moreover, we included in our analysis a comparatively small study population admitted to only one CF centre which may limit the generalizability of our results. Another limitation is the absence of lung function data for 23 patients (< 6 years) who had to be excluded from the evaluation of FEV1 in predicting personnel costs. Also, our study may not sufficiently reflect the continued trend from inpatient to outpatient and home-based therapy which should be considered when interpreting our results. When we measured resource use borne by the CF outpatient center, a trend to move from inpatient to outpatient and home-based therapy could already be observed, driven in part by economic pressures [36-39]. Although this trend towards home-based treatment especially has continued since, it has not affected the treatment patterns or amount of routine check-ups for outpatients evaluated by our study. Moreover, the low staffing level at our centre has not changed since study implementation.

## Conclusions

Our findings on the average financial requirements for health personnel provide important information for the remuneration system for CF centres. We have shown that age is a significant determinant of staff costs for CF outpatient treatment, while severity of disease and lung function did not predict cost variation. For the remuneration of outpatient services it seems therefore plausible to stratify CF patients into homogeneous cost groups according to age, and create separate reimbursement rates for two or three age groups. Given that CF centres, including ours, are substantially understaffed, the relatively low resource use for personnel identified by our study underestimates the costs needed for the provision of specialist CF outpatient care according to clinical standards. For a cost-covering reimbursement and to ensure an accurate quality of outpatient treatment it is also required to take into account additional costs due to time-consuming tasks carried out by physicians without direct patient contact. Moreover, further studies are needed to identify the further expansion of outpatient and home-based care.

## Competing interests

The authors declare that they have no competing interests. There was no funding provided for conduct of the study or preparation of the paper.

## Authors' contributions

HH had the idea for the study, designed the study, prepared the data collection, analyzed and interpreted the data, and prepared the manuscript. DS was involved in the study design, supervised the data collection, contributed to the data interpretation, and revised the manuscript critically for important intellectual content. JS made contributions to the conception of this study, analyzed and interpreted data, and revised the manuscript critically for important intellectual content. UW made substantial contributions to the implementation of the study. All authors read and approved the final draft.
